# Supplementary material for: Reframing Communication about Fall Prevention Programs to Increase Older Adults’ Intentions to Participate
Source: Int J Environ Res Public Health. 2024 May 30;21(6):704. doi: 10.3390/ijerph21060704 (PMC11203759; doi:10.3390/ijerph21060704)
Supplement: Supplementary file 1 [file ijerph-21-00704-s001.zip › Supplementary File 2.pdf]

Supplementary File 2

Pre-test survey items for the Standard flyer and Reframed flyer.

1. To what extent does this flyer emphasize the subject of ageing?
   [Original (Dutch): In hoeverre benadrukt deze flyer het onderwerp ouder worden?]

   1 = not at all [helemaal niet] -- 5 = extremely [heel sterk]

2. To what extent does this flyer evoke thoughts or feelings about ageing?
   [Original (Dutch): In hoeverre roept deze flyer gedachten of gevoelens bij u op over ouder worden?]

   1 = not at all [helemaal niet] -- 5 = extremely [heel sterk]

3. To what extent does this flyer emphasize the subject of falling?
   [Original (Dutch): In hoeverre benadrukt deze flyer het onderwerp vallen?]

   1 = not at all [helemaal niet] -- 5 = extremely [heel sterk]

4. To what extent does this flyer evoke thoughts or feelings about falling?
   [Original (Dutch): In hoeverre roept deze flyer gedachten of gevoelens bij u op over vallen?]

   1 = not at all [helemaal niet] -- 5 = extremely [heel sterk]
