# Supplementary figures and images for: Reframing Communication about Fall Prevention Programs to Increase Older Adults’ Intentions to Participate
Source: Int J Environ Res Public Health. 2024 May 30;21(6):704. doi: 10.3390/ijerph21060704 (PMC11203759; doi:10.3390/ijerph21060704)

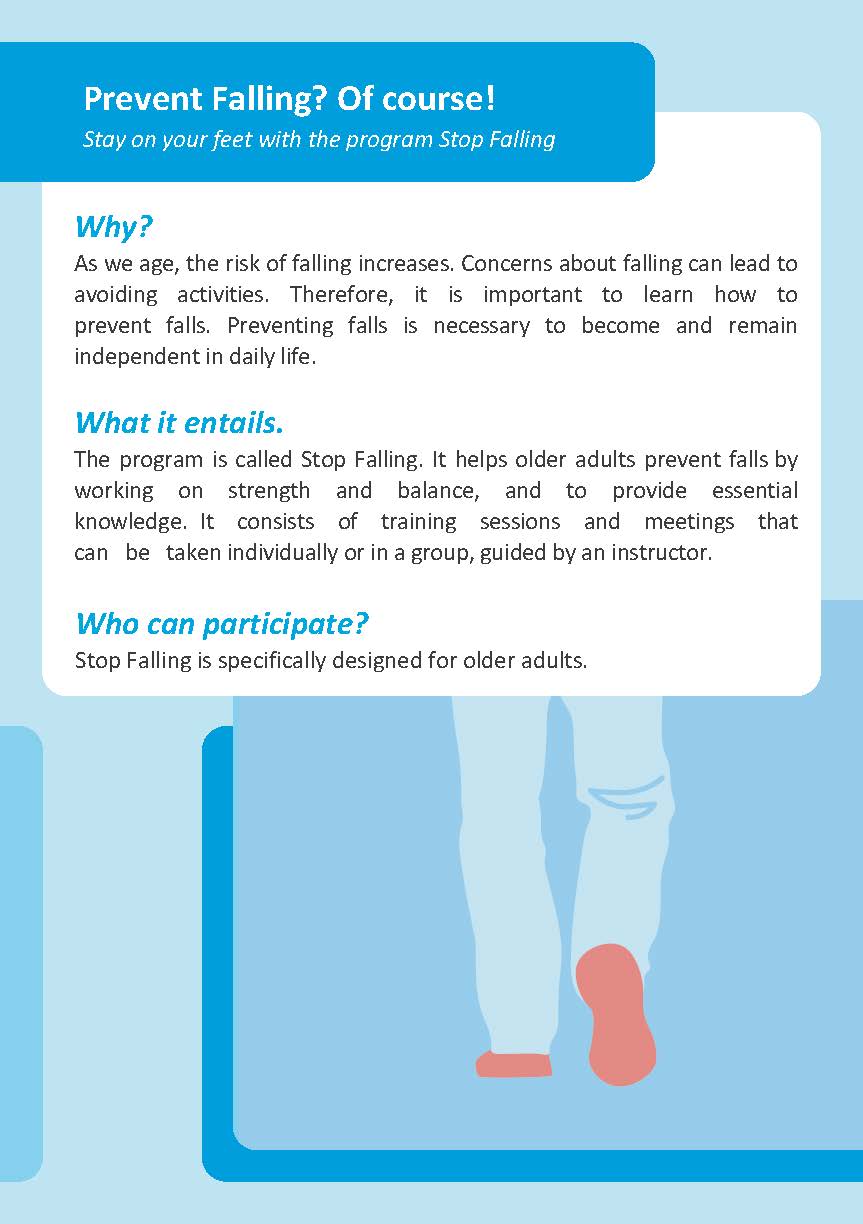

Supplement: Supplementary file 1 [file ijerph-21-00704-s001.zip › Image S1_Standard flyer Prevent Falling.jpg]

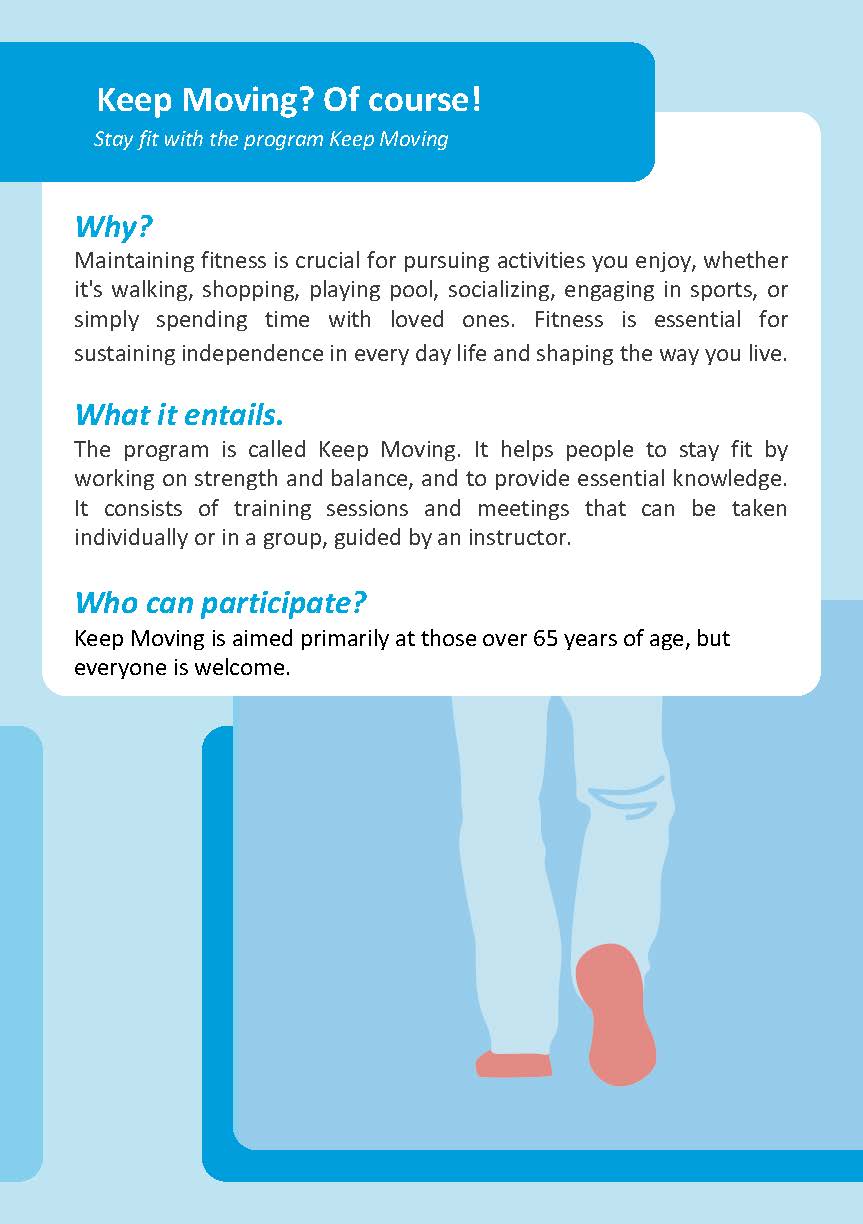

Supplement: Supplementary file 1 [file ijerph-21-00704-s001.zip › Image S2_Reframed flyer Keep Moving.jpg]
